# Collective judgment predicts disease-associated single nucleotide variants

**DOI:** 10.1186/1471-2164-14-S3-S2

**Published:** 2013-05-28

**Authors:** Emidio Capriotti, Russ B Altman, Yana Bromberg

**Affiliations:** 1 Division of Informatics, Department of Pathology, University of Alabama at Birmingham, Birmingham, AL, USA; 2 Departments of Bioengineering and Genetics, Stanford University, Stanford, CA, USA; 3 Department of Biochemistry and Microbiology, Rutgers University, New Brunswick, NJ, USA

## Abstract

**Background:**

In recent years the number of human genetic variants deposited into the publicly available databases has been increasing exponentially. The latest version of dbSNP, for example, contains ~50 million validated Single Nucleotide Variants (SNVs). SNVs make up most of human variation and are often the primary causes of disease. The non-synonymous SNVs (nsSNVs) result in single amino acid substitutions and may affect protein function, often causing disease. Although several methods for the detection of nsSNV effects have already been developed, the consistent increase in annotated data is offering the opportunity to improve prediction accuracy.

**Results:**

Here we present a new approach for the detection of disease-associated nsSNVs (Meta-SNP) that integrates four existing methods: PANTHER, PhD-SNP, SIFT and SNAP. We first tested the accuracy of each method using a dataset of 35,766 disease-annotated mutations from 8,667 proteins extracted from the SwissVar database. The four methods reached overall accuracies of 64%-76% with a Matthew's correlation coefficient (MCC) of 0.38-0.53. We then used the outputs of these methods to develop a machine learning based approach that discriminates between disease-associated and polymorphic variants (Meta-SNP). In testing, the combined method reached 79% overall accuracy and 0.59 MCC, ~3% higher accuracy and ~0.05 higher correlation with respect to the best-performing method. Moreover, for the hardest-to-define subset of nsSNVs, *i.e*. variants for which half of the predictors disagreed with the other half, Meta-SNP attained 8% higher accuracy than the best predictor.

**Conclusions:**

Here we find that the Meta-SNP algorithm achieves better performance than the best single predictor. This result suggests that the methods used for the prediction of variant-disease associations are orthogonal, encoding different biologically relevant relationships. Careful combination of predictions from various resources is therefore a good strategy for the selection of high reliability predictions. Indeed, for the subset of nsSNVs where all predictors were in agreement (46% of all nsSNVs in the set), our method reached 87% overall accuracy and 0.73 MCC. Meta-SNP server is freely accessible at http://snps.biofold.org/meta-snp.

## Introduction

The most common form of human genetic variation is single nucleotide polymorphisms (SNVs) [1]. Trivially, non-coding region SNVs are more common than coding. However, fewer, percentage-wise, non-coding variants have thus far been characterized as disease-causing than coding, non-synonymous SNVs (nsSNVs; *e.g*. HGMD [2]). This fact is likely the result of experimentally difficult and therefore limited exploration into the non-coding world. Whatever the reason, however, most of the existing computational tools study the effects of nsSNVs specifically [3-6].

Many human diseases are monogenic, *i.e*. caused by damage to a single gene [7]. Identifying SNVs causative of monogenic disease is fairly straightforward. These are always functionally disruptive and consistently present in the disease population, but less frequently in healthy controls [8]. Complex diseases, on the other hand, are generally caused by a combination of moderately deleterious mutations in different genes; often leading to a disruption of the broader functional networks involved. Any one of these SNVs is unlikely to be significantly visible in the overarching background of human variation [9,10].

In the last decade, several algorithms have been developed to predict disease-related and functionally deleterious variants [11-21]. Methods that aim to identify disease-associated nsSNVs (which cause single amino acid substitutions in the protein sequence) are a different set of tools from those that look for nsSNVs that disrupt protein molecular function [11,17,21]. The latter focus on a single protein, a hard enough task in itself, while the former need to identify the mutation effect on the phenotype of an entire organism. One of the biggest challenges facing the nsSNV-disease mapping methods is the collection of development/testing data sets; *i.e*. beyond monogenic disease mutations and coding variants found by genome-wide association studies (GWAS), the experimental identification of complex disease-associated mutations is very subjective. The majority of the current methods rely on the manually curated collections of disease-associated variants from OMIM [7], SwissVar [22], and, more recently, the dbSNP [23] clinical SNV collections. Once the data sets are collected, all methods use some combination of the affected protein sequence/structure features and functional annotations to look for patterns indicative of disease involvement.

The overlap in development data sets and features of interest suggests that most methods should "pick up" similar patterns in the data. However, recent estimates [3] show that different tools vary significantly in the predictions they make, while each still attaining relatively high levels of accuracy. Method orthogonality, *i.e*. each one method getting a different set of variants right, may be one explanation to this phenomenon.

Given the relative lack of new annotated data sets and the high levels of accuracy already attained, moving the field forward has been difficult. Newly developed methods at best boast improved annotation speeds or incremental gains in performance, often at cost of limited applicability. In this study we aimed to take advantage of method orthogonality to complement each tool's predictions with those of the other tools. The meta-predictor that we developed (Meta-SNP) identifies disease-causing nsSNVs by coupling some of the leading methodologies in prediction of nsSNV-disease (PhD-SNP [16]) and nsSNV-function associations (PANTHER [18], SIFT [17], SNAP [11]).

## Methods

### Dataset and benchmarking

Training and testing machine learning approaches require appropriate representative set of reliably annotated data. To develop a method for the detection of disease-associated nsSNVs we needed a large set of well-annotated disease-related (positive cases) and polymorphic (negative cases) variants. Although for Mendelian disease the annotation of disease-causing variations is reliable, the selection of polymorphic nsSNVs is still a problem. In this work, we consider as disease and polymorphic variants those annotated in SwissVar [22] as Disease and Polymorphism, respectively. Note that the SwissVar Polymorphisms may (and probably do) still carry undiscovered disease associations.

Our training set is composed of disease-related and polymorphism variants from the SwissVar database October 2009 release (*SV-2009*). All methods were also tested on an independent set of protein variations from a newer version of SwissVar (February 2012 release). The *SV-2009 *dataset consists of 35,766 nsSNVs from 8,667 proteins. To build this set we (1) extracted from SwissVar all variants that were not annotated as Unclassified and only those whose annotations did not change between the 2009 and 2012 and (2) balanced the number of disease-associated and polymorphic mutations by taking all disease variants (17,883 variants) and randomly selecting an equal sized sets of polymorphsims. The variants added to SwissVar between 2009 and 2012 included 4,387 polymorphisms and 486 disease-associated mutations (excluding variants in proteins from SV-2009). Of these, the *NSV-2012 *data set is a disease/polymorphism-balanced subset that consists of 972 nsSNVs (all 486 disease variants and a randomly selected set of 486 polymorphisms) from 577 proteins that were not found in SV-2009.

Both *SV-2009 *and *NSV-2012 *datasets were partitioned into three subsets according to the agreement in predictions returned by the four algorithms (see *Prediction methods *and Supplementary Online Material, Table S1). The *Consensus *subset consists of the variations for which all four predictors returned identical predictions (46% of SV-2009 and 42% of NSV-2012). The *Tie *subset is the set of variants that were classified as disease-related and polymorphic by equally many predictors, two for each classification (14%/16%). The *Majority *is the subset of nsSNVs where three predictors agreed in the judgment and one disagreed (40%/42%).

### Prediction methods

In this work we predicted the effect of nsSNVs using PANTHER, PhD-SNP, SIFT and SNAP. Note that PANTHER, SIFT, and SNAP annotate variants as disruptive of protein function or equivalent to wild-type, while PhD-SNP particularly recognizes disease-associated substitutions.

PhD-SNP is a Support Vector Machines (SVMs) based method trained to predict disease-associated nsSNVs using sequence information. The methods takes as input the information about the mutation, such as its sequence environment and profile at the mutated site, calculated by BLASTing [24] it against the UniRef90 database [25]. For each mutation, PhD-SNP returns an output score (ranged 0-1) that represents the probability of this nsSNV being associated with disease. The method considers 0.5 to be the threshold above which the nsSNVs are predicted to be disease-associated.

The PANTHER algorithm is based on a library of Hidden Markov Models (HMMs) obtained from the multiple sequence alignments of different protein families. PANTHER predicts the effect of nsSNVs in a two-step procedure. First, the affected protein is compared to all HMMs in the library to find the HMM of the query protein family. Then, this HMM is used to calculate the probability of the nsSNV disrupting the function of the affected protein. Note that when PANTHER is not able to map the affected protein to one of the families in its library, no output is returned.

SIFT uses evolutionary information to make predictions with regard to functional effects of nsSNVs. Our local installation of SIFT used the UniRef90 database for the necessary PSI-BLASTs. SIFT scores are normalized to range 0-1, where any score >0.05 represents a neutral substitution, while mutations scoring <0.05 are functionally deleterious.

SNAP is a neural network-based method that takes as input the biochemical features of the given substitution as well as predicted protein structural and functional features to differentiate neutral and non-neutral variants. The local installation of SNAP produces a raw score of -100 to 100, where all predictions >0 are non-neutral and <0 are neutral. Note the raw score is converted into a reliability index for all web-based predictions.

### Implementation of the Meta-SNP algorithm

We trained Meta-SNP, a random forest-based binary classifier to discriminate between disease-related and polymorphic non-synonymous SNVs. Meta-SNP takes as input the output of the four predictors described above as an eight-element feature vector composed of two groups of four elements each. The first group is the set of raw output scores of the variant predictions from PANTHER, PhD-SNP, SIFT and SNAP. In case one of the input methods does not return a prediction, we used the method-defined default threshold for differentiating neutrals and non-neutrals as input to Meta-SNP (SNAP = 0, SIFT = 0.05, PhD-SNP = 0.5, PANTHER = 0.5).

The second group contains four elements extracted from the PhD-SNP protein sequence profile: (1 and 2) frequencies of the wild-type (F_wt_) and mutant (F_mut_) residues in the mutated site, (3) the total number of sequences aligned at the mutated site (N_al_) and (4) the conservation index (CI) [26]. Sequence profile information modulates Meta-SNP predictions by the conservation of the mutated position. This information is redundant across the four component methods, so for Meta-SNP we used only one version of the sequence profile - that from PhD-SNP.

Meta-SNP is a 100-tree RandomForest WEKA [27] library implementation, trained on *SV-2009 *using 20-fold cross-validation. The predictor outputs the probability that a given nsSNV is disease-related, where scores >0.5 indicate that the given the variant is disease-causing.

### Measures of performance

In all measures of performance (assuming that positives indicate disease and negatives indicate polymorphisms), TP (true positives) are correctly predicted disease-associated variants, TN (true negatives) are correctly predicted polymorphisms, FP (false positives) polymorphic variants annotated as disease-causing, and FN (false negatives) are disease-associated variants predicted to be polymorphic.

Predictor performance was evaluated using the following metrics: positive and negative predicted values (respectively PPV and NPV), true positive and negative rates (respectively TPR and TNR), and overall accuracy (Q_2_; Eqn. 1)

(1) $$\begin{array}{c} PPV=\frac{TP}{TP+FP}\;TPR=\frac{TP}{TP+FN}\\ NPV=\frac{TN}{TN+FN}\;TNR=\frac{TN}{TN+FP}\\ Q_{2}=\frac{TP+TN}{TP+FP+TN+FN} \end{array}$$

We also computed the Matthew's correlation coefficient MCC (Eqn. 2) as:

(2) $$MCC\left(s\right)=\frac{TP\times TN-FP\times FN}{\sqrt{\left(TP+FP\right)\left(TP+FN\right)\left(TN+FP\right)\left(TN+FN\right)}}$$

For each prediction, the binary classification (disease/polymorphism) is made at the output threshold of 0.5. Thus, if probability of disease classification, *P(D)*, is >0.5 the mutation is predicted to be disease associated. If *P(D) *≤0.5, the variant is predicted to be polymorphic. A reliability index (RI) for all predictions is calculated as follows:

(3) $$RI=20\times abs\left[P\left(D\right)-0.5\right]$$

Thus, RI is ranged 0-10 for both negative (polymorphic) and positive (disease-associated predictions). Varying RI threshold for annotating variants allows trading off accuracy of predictions for the coverage of all disease-associated and polymorphic variants in any given set.

We also report the area under the receiver operating characteristic (ROC) curve (AUC), calculated by plotting the True Positive Rate (positive sensitivity) as a function of the False Positive Rate (1-negative sensitivity) at different probability thresholds of annotating a variant as disease-associated or polymorphic. All the same metrics (Eqn. 1 and 2) were used to calculate the pairwise similarities between predictors on the subsets of variants predicted by both methods.

## Results

### Performances of four available methods

First we tested the accuracy of four stand-alone methods, PANTHER, PhD-SNP, SIFT and SNAP, on a large dataset of nsSNVs (*SV-2009*; Tables 1 and Additional File 1 Table S2). For this set, PANTHER and PhD-SNP are most accurate, reaching ~75% overall accuracy (Eqn. 1) and ~0.83 AUC (SIFT 70%/0.73, SNAP 64%/0.79 Q2/AUC, respectively). Note that due to the lack of the appropriate number of homologous sequences SIFT and PANTHER did not return any predictions in 8% and 26% of the cases, respectively. Also note that even though the PhD-SNP results are obtained with a 20-fold cross-validation procedure, its performance estimates may be biased as it was trained on the *SV-2009 *dataset. SNAP's lower Q_2 _value at high AUC suggests many false positive predictions. This is an expected outcome, as not all functionally deleterious mutations are disease associated [8,28,29].

**Table 1:** Component method performance

Method	Q_2_	PPV	TPR	NPV	TNR	MCC	AUC	%DB
PANTHER	0.74	**0.79**	0.73	0.69	0.74	0.48	0.82	74
PhD-SNP	**0.76**	0.78	0.74	0.75	**0.78**	**0.53**	**0.84**	100
SIFT	0.70	0.74	0.64	0.68	0.76	0.41	0.73	92
SNAP	0.64	0.59	**0.90**	**0.79**	0.38	0.33	0.79	100

Q_2_=Overall accuracy, PPV and NPV=Positive and Negative Predicted Values, TPR and TNR=True Positive and Negative Rates. MCC=Mathew's correlation, AUC=area under the (ROC) curve, %DB is the fraction of the SV-2009 dataset for which a prediction is returned.

### Scoring consensus predictions

**Figure 1:**
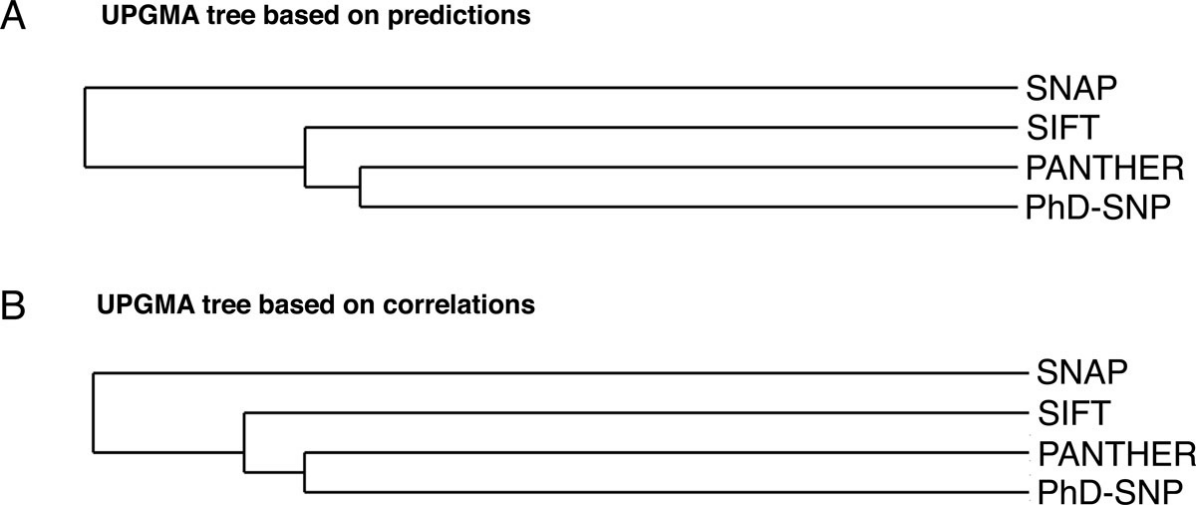
**Illustrating orthogonality of the component methods**. Unweighted Pair Group Method with Arithmetic Mean (UPGMA) trees visualize the similarity between PANTHER, PhD-SNP, SIFT and SNAP according to the overlap (panel A) and the correlation (panel B) between the predictions in Table 2. The trees were drawn using the drawtree package [31].

We first analyzed the similarities between PANTHER, PhD-SNP, SIFT and SNAP by calculating percentage consensus predictions and the correlation between all possible pairs of methods (Table 2). These values were used to visualize the similarities between the methods with two Unweighted Pair Group Method with Arithmetic Mean (UPGMA) trees (Figure 1). PANTHER and PhD-SNP algorithms returned the highest number of common predictions (76%, MCC = 0.52). On the other hand, PhD-SNP and SNAP had only 64% of the predictions in common (correlation 0.36).

**Table 2:** Component method prediction "distance"

	PANTHER	PhD-SNP	SIFT	SNAP
PANTHER	-	0.52	0.49	0.39
PhD-SNP	0.76	-	0.45	0.36
SIFT	0.74	0.73	-	0.38
SNAP	0.68	0.64	0.65	-

Similarities between pairs of predictors calculated on the SV-2009 dataset. The fraction of consensus predictions is reported at the bottom of the diagonal and the correlation up of the diagonal.

In addition, we evaluated the accuracy of the PhD-SNP on *Consensus, Majority *and *Tie *subset of predictions (see *Datasets and Benchmarking*). We expected a decrease in level of accuracy from the set of variants where all methods agree in their predictions (*Consensus*) to those where methods largely disagree (*Tie*). This hypothesis is confirmed (Table 3) with the overall accuracy and MCC of PhD-SNP decreasing for these two from 87% to 61% and 0.73 to 0.16, respectively. An intermediate level of accuracy is attained on the *Majority *subset, where most of the predictors agree (70% Q_2 _and 0.37 MCC).

**Table 3:** Performances of the PhD-SNP and Meta-SNP on training set

Dataset (% of SV-2009)	Tool	Q_2_	PPV	TPR	NPV	TNR	MCC	AUC
*SV-2009* (100%)	PhD-SNP	0.76	0.78	0.74	0.75	0.78	0.53	0.84
	Meta-SNP	**0.79**	**0.80**	**0.79**	**0.79**	**0.80**	**0.59**	**0.87**
*Consensus* (46%)	PhD-SNP	0.87	0.87	0.92	0.87	0.79	0.73	0.89
	Meta-SNP	0.87	**0.88**	0.92	0.87	**0.80**	0.73	**0.91**
*Majority* (40%)	PhD-SNP	0.70	0.67	0.56	0.72	0.80	0.37	0.77
	Meta-SNP	**0.75**	**0.72**	**0.64**	**0.76**	**0.82**	**0.47**	**0.82 **
*Tie* (14%)	PhD-SNP	0.61	0.51	0.43	0.66	0.73	0.16	0.67
	Meta-SNP	**0.69**	**0.62**	**0.57**	**0.73**	**0.76**	**0.34**	**0.75**

Q_2_=Overall accuracy, PPV and NPV=Positive and Negative Predicted Values, TPR and TNR=True Positive and Negative Rates. MCC=Mathew's correlation, AUC=area under the (ROC) curve.

To understand the difference in the performance achieved on the three subsets (*Consensus, Majority and Tie*) we evaluated the residue conservation in the mutated positions using the protein sequence profile calculated by PhD-SNP BLAST run (see Prediction methods). We compared the distributions of the wild-type and mutant residue frequencies (respectively F_wt _and F_mut_) in the mutated positions for disease-related and polymorphic nsSNVs. In addition, we analyzed the differences in the distributions of the conservation index (CI). Our results show (Figure 3 and Table S3) increasing overlap between the distributions of F_wt_, F_mut _and CI for disease-related and polymorphic nsSNVs from the *Consensus *(Figure 3D,E,F) to the *Majority *(Figure 3G,H,I) to the *Tie *(Figure 3J,K,L) subset. As expected, an intermediate (average) distribution difference is observed for the whole *SV-2009 *dataset (Figure 3A,B,C).

**Figure 2:**
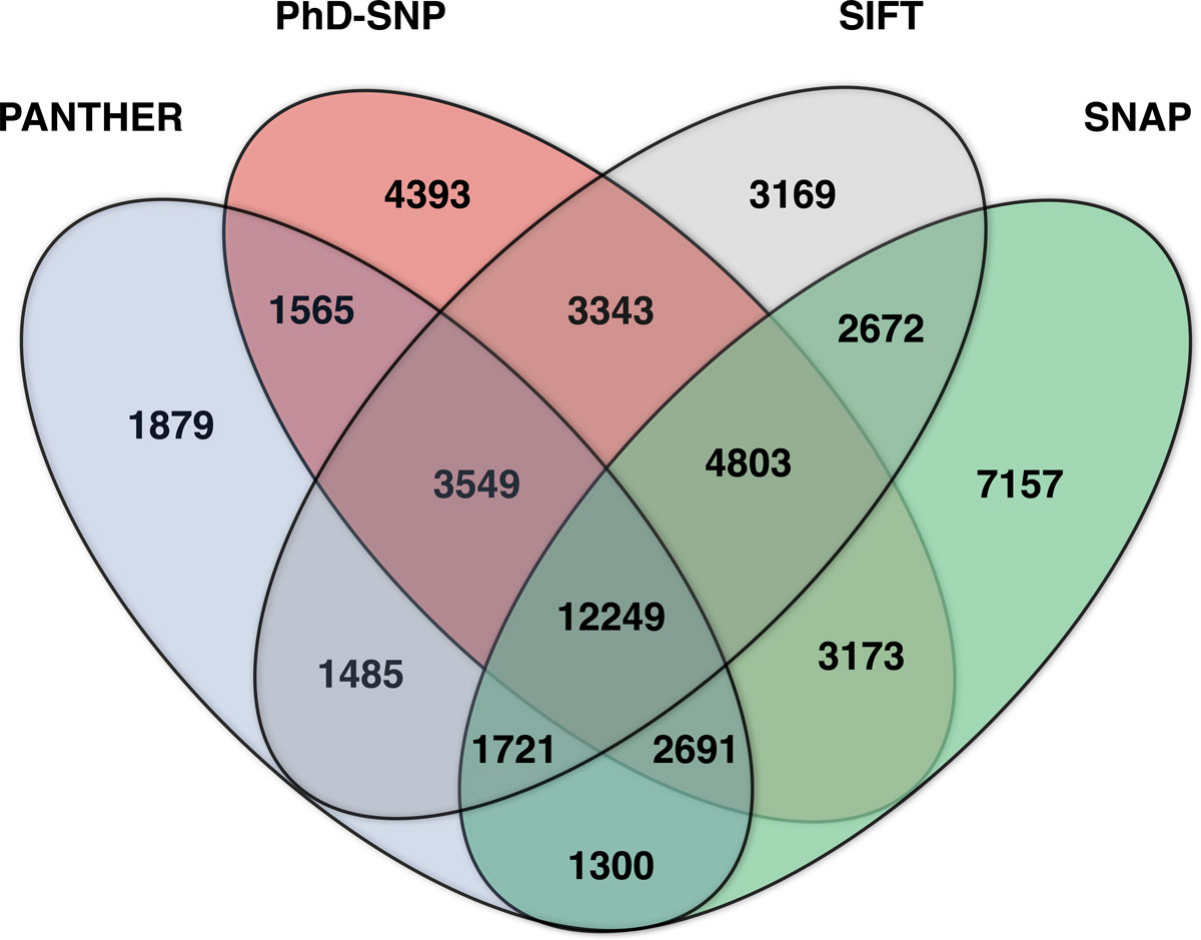
**Venn diagram of prediction overlaps**. Overlap between the predictions returned by PANTHER (blue), PhD-SNP (red), SIFT (grey) and SNAP (green), generated using Venny [32].

**Figure 3:**
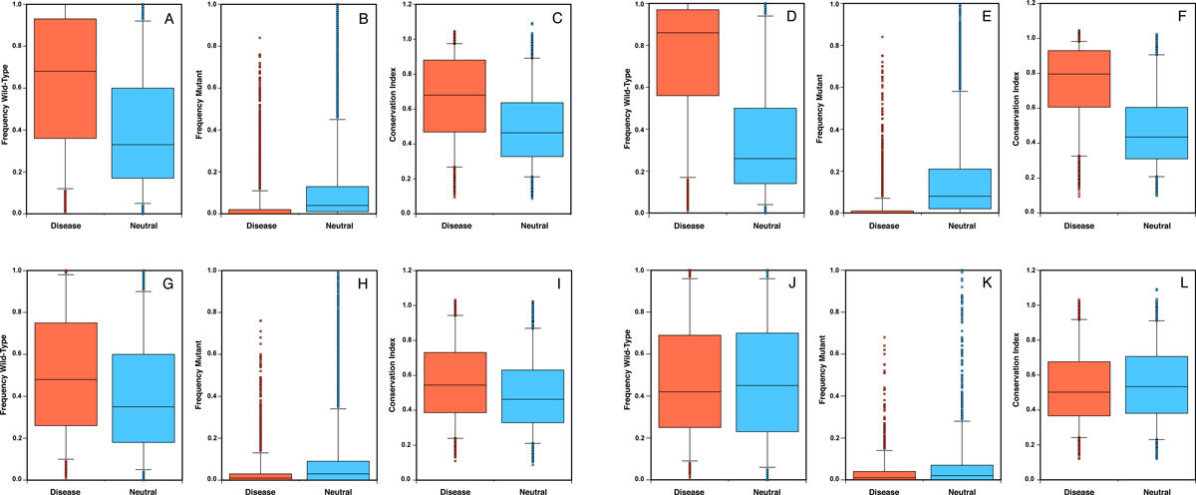
**The overlap in sequence profile-based feature distributions is most visible for hardest to predict set of variants**. Distributions of the frequencies of the wild-type (F_wt_) and mutant (F_m_) residues and conservation indices (CI) for disease-related (red) and polymorphic (blue) nsSNVs were computed from sequence profiles. The distributions are calculated on *SV-2009 *dataset (panels A, B, C) and its subsets: *Consensus *(panels D, E, F), *Majority *(panels G, H, I) and *Tie *(panels J, K, L). Distributions of all profile features overlap most for *Tie *set and least for *Consensus *set.

### Performances of the meta-predictor

**Figure 4:**
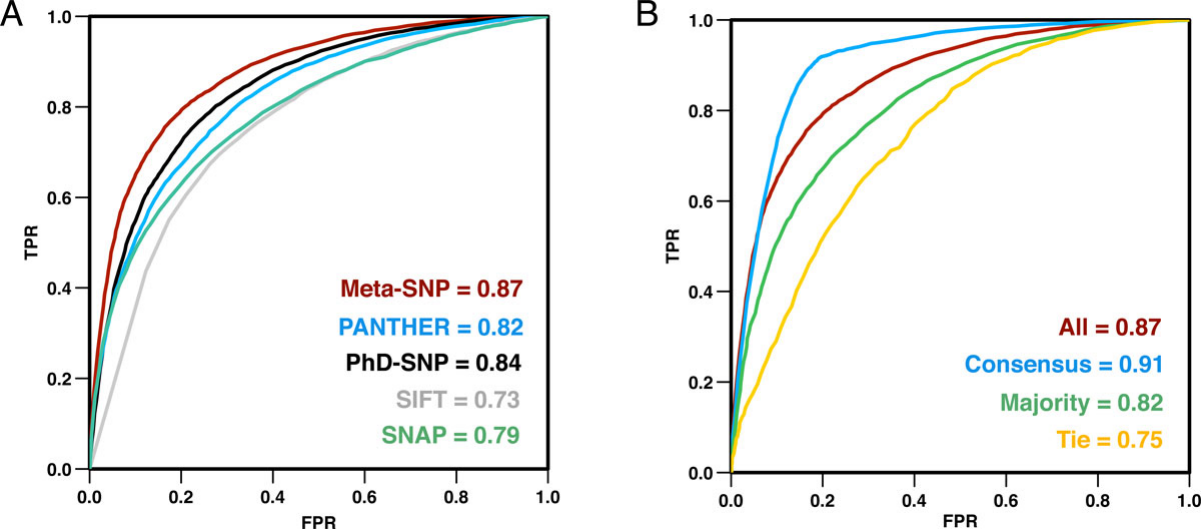
**Meta-SNP is more accurate in predicting disease-associated nsSNVs than all of its components for all data sets**. **(A) **Receiver operating characteristic (ROC) curves for all the prediction algorithms show that Meta-SNP is a better predictor than all of its component methods. **(B) **Of all subsets of SV-2009, however, Meta-SNP performs best on the *Consensus *set, followed by *Majority *and *Tie *subsets.

To improve the detection of deleterious variants, we developed a meta-predictor (Meta-SNP) that combines the outputs of PANTHER, PhD-SNP, SIFT and SNAP. Meta-SNP uses single predictor outputs as in input; it was trained and tested on the *SV-2009 *dataset using a 20-fold cross-validation procedure. Meta-SNP reaches 79% overall accuracy, 0.59 MCC and 0.87 AUC (Table 4). While Meta-SNP outpredicts all other methods for all data sets, an accuracy decrease from the *Consensus *to the *Majority *to the *Tie *subset is still observed (87%, 75%, 69% Q_2_, respectively). The AUC for Meta-SNP is also higher than that of the single methods for all of the *SV-2009 *subsets (Figure 4).

**Table 4:** Performances of the component methods and Meta-SNP on testing set

Method	Dataset	Q_2_	PPV	TPR	NPV	TNR	MCC	AUC	%DB
PANTHER	NSV-2012	0.74	0.81	0.71	0.68	0.78	0.49	0.75	75
PhD-SNP		0.77	0.78	0.77	0.77	0.78	0.55	0.84	100
SIFT		0.68	0.79	0.53	0.62	0.85	0.39	0.73	93
SNAP		0.64	0.59	0.91	0.80	0.38	0.34	0.79	100
CONDEL		0.75	0.78	0.70	0.72	0.81	0.51	0.82	100
Meta-SNP	NSV-2012	0.79	0.79	0.80	0.80	0.79	0.59	0.86	100
	*Consensus*	0.87	0.88	0.89	0.87	0.85	0.74	0.91	42
	*Majority*	0.77	0.77	0.74	0.77	0.79	0.53	0.83	42
	*Tie*	0.68	0.61	0.67	073	0.69	0.35	0.72	16

Q_2_=Overall accuracy, PPV and NPV=Positive and Negative Predicted Values, TPR and TNR=True Positive and Negative Rates. MCC=Mathew's correlation, AUC=area under the (ROC) curve. %DB is the fraction of the NSV-2012 dataset for which a prediction is returned.

**Figure 5:**
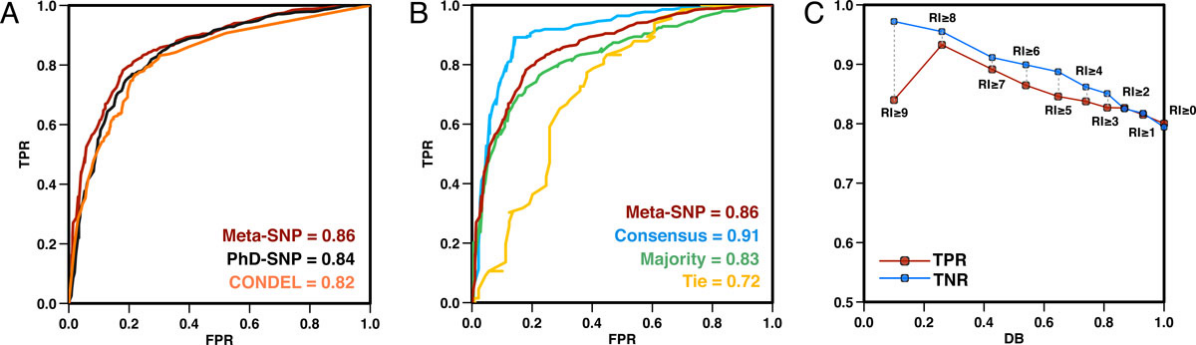
**Comparison between Meta-SNP, CONDEL and PhD-SNP**. **(A) **Performances of **CONDEL, PhD-SNP and Meta-SNP **on the NSV-2012 dataset. **(B) **Meta-SNP accuracy on NSV-2012 dataset and its three subsets. **(C) **Accuracy of Meta-SNP in terms of TPR and TNR improves as a function of increasing. Note that there are only 25 disease causing variants at RI = 9, resulting in an artifact of the curve - an unexpected drop in accuracy, RI. TPR, TNR and RI are defined in Methods. DB is the fraction of the NSV-2012 dataset with an RI higher or equal than a given threshold.

Meta-SNP was additionally tested on *NSV-2012*, a disease/polymorphism balanced subset of nsSNVs added to SwissVar from October 2009 to February 2012 and belonging to proteins not found in *SV-2009 *(see Methods). The results on this dataset confirm that Meta-SNP performs better than PhD-SNP and all other predictors (see Figure 5A, Tables 4 and Additional File 1 Table S4). Note Meta-SNP performance on the whole set of variants added to SwissVar 2009-2012 is similar in overall accuracy and AUC to that achieved on the disease/polymorphism balanced NSV-2012 dataset, albeit, as expected, at a lower PPV.

We also compared the performance of Meta-SNP to that of CONDEL [30], another recently developed meta-predictor. Meta-SNP is 4% more accurate (Q_2_) than CONDEL and achieves higher AUC (Table 4). As expected, the Meta-SNP prediction accuracy still drops between the *Consensus, Majority *and *Tie *subsets of *NSV-2012 *(Q_2_: 87% to 77% to 68%, AUC: 0.91 to 0.83 to 0.72, respectively, see Table S4 and Figure 5B). Finally, the Meta-SNP reliability index (see RI in Methods) helps selecting more accurate predictions (Figure 5C); *e.g.*, the *NSV-2012 *predictions with RI≥5 are on average ~87% accurate, albeit at the cost to recall (only~65% of the variants reach this score). Similar trends are observed for the *Consensus, Majority and Tie *subsets (Additional File 1 Figure S1).

## Discussion

The results presented in this work show that combining predictors of nsSNV effects into a single unique meta-predictor (Meta-SNP) improves the detection of disease-causing variants. The Meta-SNP algorithm performs slightly better (3% gain in accuracy, Q_2_) than PhD-SNP, the best of the component methods for picking disease-associations. Although this improvement can not be considered very high, the advantage of Meta-SNP over a single predictor is three-fold: (1) the use of four orthogonal methods makes Meta-SNP more robust to handling new data sets, which may not follow the same distribution as sets used for method development, (2) Meta-SNP produces a single score, rather than four separate scores, for the prediction of disease-related nsSNVs and (3) Meta-SNP significantly outperforms all component methods in classifying the mutations, which are "border-line", *i.e*. ones that are very difficult to classify as disease-associated or polymorphic with current computational means.

As our observations (in Figure 3 and Additional File 1 Table S3) suggest, the overlap between distributions of evolutionary features of disease and polymorphic variants, especially for the *Tie *data set, may indicate either (1) the lack of resolution in experimental data (*i.e*. polymorphisms may actually be disease causing mutations, which have not yet identified as such), (2) inaccuracies in building evolutionary profiles (i.e. simple PSI-BLAST searches may not be enough for all cases), or (3) our inability to differentiate variants contributing to complex disease phenotypes (i.e. when more than one variant is necessary for the disease phenotype to become visible). In all of these cases, however, the computational algorithms that strongly rely on a single evolutionary model are unable to differentiate disease variants from polymorphisms. While the calculation of accurate alignments and profiles is key to the performance of the predictive methods, we should also focus on improving resolution of our experimental annotations and available data collections. Additionally, understanding the contribution of multiple correlated nsSNVs in one or many proteins will enable discrimination between disease-associated and polymorphic variants in unconserved sites. In the mean time, combining many methods into a single model, Meta-SNP, provides a new and significantly more accurate way of assessing disease-association of human variants, most often mis-predicted by single sequence-based methods.

## Conclusion

We developed a meta-predictor (Meta-SNP) that integrates the PANTHER, PhD-SNP, SIFT and SNAP methods to predict disease-associated nsSNVs. To quantify the increase in accuracy achieved by the combination of the different methods we compared the performance of our meta-predictor against that reached by the single methods. Using a balanced set of 35,766 nsSNVs, the meta-predictor attains ~3% higher accuracy, 0.03 higher AUC and 0.06 higher MCC with respect to PhD-SNP, the highest scoring of all stand-alone predictors. Although this overall increase in performance is not high, the performance is significantly improved on the ~58% of the dataset where the component predictors disagree (*Majority *and *Tie *subsets). For these subsets the meta-predictor achieved ~6% higher overall accuracy and 0.12 higher MCC with respect to PhD-SNP. Meta-SNP is robust for new data as it reached similar levels of accuracy on a set of 972 new nsSNVs in proteins not included in the initial training dataset.

## Abbreviations used

Single Nucleotide Polymorphism: SNP; single nucleotide variant: SNV; nsSNV: non-synonymous single nucleotide variant; Q_2_: overall accuracy; TPR and TNR: true positive and negative rates; PPV and NPV: positive and negative predictive values; MCC: Matthews Correlation Coefficient; RI: Reliability Index.

## Competing interests

The authors declare they have no conflict of interests in relation to the SNP-SIG issue article.

## Authors' contributions

EC, RBA and YB conceived this work and participated in its design. EC implemented the Meta-SNP algorithm. EC and YB ran the predictions of the single methods and wrote the manuscript. All authors read and approved the manuscript. This paper has been Edited by Sean Mooney, Buck Institute, Novato (CA).

## Supplementary Material

Additional file 1 Collective judgment predicts disease-associated single nucleotide variants.
Table S1. Composition of the datasets.
Table S2. Performance of the four methods on the SV-2009 subsets.
Table S3. Comparison of the distribution of sequence profile features.
Table S4. Performances of the four methods on the NSV-2012 subsets.
Fig. S1. Performance Meta-SNP as a function of the RI. Click here for file

Additional file 2 SV-2009.txt: SV-2009 dataset Click here for file

Additional file 3 NSV-2012.txt: NSV-2012 dataset Click here for file

## Declarations

The publication costs for this article were funded by the above grants supporting EC and YB.

This article has been published as part of *BMC Genomics *Volume 14 Supplement 3, 2013: SNP-SIG 2012: Identification and annotation of SNPs in the context of structure, function, and disease. The full contents of the supplement are available online at http://www.biomedcentral.com/bmcgenomics/supplements/14/S3
