# Supplementary material for: Collective judgment predicts disease-associated single nucleotide variants
Source: BMC Genomics. 2013 May 28;14(Suppl 3):S2. doi: 10.1186/1471-2164-14-S3-S2 (PMC3839641; doi:10.1186/1471-2164-14-S3-S2)
Supplement: Additional file 1 — Collective judgment predicts disease-associated single nucleotide variants. Table S1. Composition of the datasets. Table S2. Performance of the four methods on the SV-2009 subsets. Table S3. Comparison of the distribution of sequence profile features. Table S4. Performances of the four methods on the NSV-2012 subsets. Fig. S1. Performance Meta-SNP as a function of the RI. [file 1471-2164-14-S3-S2-S1.PDF]

SUPPLEMENTARY MATERIAL

Collective judgment predicts disease-associated single nucleotide variants

Emidio Capriotti[1], Russ B Altman[2], Yana Bromberg[3*].*

[1] Division of Informatics, Department of Pathology, University of Alabama at Birmingham, Birmingham (AL), United States of America.

[2]Departments of Bioengineering and Genetics, Stanford University, Stanford (CA), United States of America.

[3] Department of Biochemistry and Microbiology, Rutgers University, New Brunswick (NJ), United States of America.

*Corresponding author: Emidio Capriotti, emidio@uab.edu, Yana Bromberg, yanab@rci.rutgers.edu.

Table S1. Composition of the datasets

	Proteins	Variants	*Consensus* (All/Disease)	*Majority* (All/Disease)	*Tie* (All/Disease)
SV-2009	8,667	35,766	16,383/9,879	14,258/5,955	5,125/2,049
NSV-2012	577	972	408/222	409/198	155/66
Total (SV-2012)	9,244	36,738	16,791/10,101	14,667/6,153	5,280/2,115

Table S2. Performance of the four methods on the SV-2009 subsets

Method	Subset	Q_2	PPV	TPR	NPV	TNR	MCC	%DB
PANTHER	*Consensus*	0.88	0.89	0.93	0.85	0.77	0.72	35
PhD-SNP		0.87	0.87	0.92	0.87	0.79	0.73	46
SIFT		0.87	0.88	0.92	0.86	0.80	0.73	43
SNAP		0.87	0.87	0.92	0.87	0.80	0.73	46
PANTHER	*Majority*	0.65	0.66	0.53	0.64	0.75	0.29	27
PhD-SNP		0.70	0.67	0.56	0.72	0.80	0.37	40
SIFT		0.59	0.51	0.40	0.62	0.72	0.13	39
SNAP		0.47	0.43	0.88	0.66	0.17	0.07	40
PANTHER	*Tie*	0.53	0.51	0.38	0.54	0.67	0.05	6
PhD-SNP		0.61	0.51	0.43	0.66	0.73	0.16	14
SIFT		0.47	0.41	0.29	0.50	0.63	-0.08	11
SNAP		0.39	0.39	0.87	0.46	0.07	-0.09	14

Q_2=Overall accuracy, PPV and NPV=Positive and Negative Predicted Values, TPR and TNR=True Positive and Negative Rates. MCC=Mathew's correlation. %DB is the fraction of the SV-2009 dataset for which a prediction is returned.

Table S3. Comparison of the distribution of sequence profile features

Dataset	Frequency Wild-Type				Frequency Mutant				Conservation Index			
	M[D]	M[P]	d	p	M[D]	M[P]	d	p	M[D]	M[P]	d	p
SV-2009	0.68	0.33	0.32	0	0.00	0.04	0.43	0	0.68	0.46	0.30	0
Consensus	0.86	0.26	0.54	0	0.00	0.08	0.63	0	0.80	0.43	0.50	0
Majority	0.48	0.35	0.14	0	0.01	0.03	0.28	0	0.54	0.46	0.13	0
Tie	0.41	0.45	0.04	0.06	0.01	0.02	0.16	0	0.50	0.53	0.06	2×10^{-4}

D=disease-related, P=polymorphic. M is the average valued of the distribution. p and d are the p-value and the distance between the distributions of the values for disease-related and neutral class obtained using the Kolmogorov-Smirnov test.

Table S4. Performances of the four methods on the NSV-2012 subsets

Method	Subset	Q_2	PPV	TPR	NPV	TNR	MCC	%DB
PANTHER	*Consensus*	0.88	0.90	0.89	0.85	0.87	0.76	30
PhD-SNP		0.87	0.86	0.89	0.87	0.83	0.73	42
SIFT		0.88	0.89	0.89	0.87	0.86	0.75	38
SNAP		0.87	0.86	0.89	0.87	0.83	0.73	42
PANTHER	*Majority*	0.70	0.75	0.68	0.65	0.72	0.40	32
PhD-SNP		0.76	0.77	0.71	0.75	0.80	0.51	42
SIFT		0.56	0.63	0.22	0.54	0.88	0.13	40
SNAP		0.51	0.49	0.92	0.61	0.12	0.06	42
PANTHER	*Tie*	0.31	0.28	0.13	0.32	0.55	-0.36	12
PhD-SNP		0.60	0.53	0.55	0.66	0.64	0.19	16
SIFT		0.19	0.03	0.01	0.25	0.43	-0.63	12
SNAP		0.38	0.40	0.85	0.09	0.01	-0.26	16

Q_2=Overall accuracy, PPV and NPV=Positive and Negative Predicted Values, TPR and TNR=True Positive and Negative Rates. MCC=Mathew’s correlation. %DB is the fraction of the NSV-2012 dataset for which a prediction is returned.

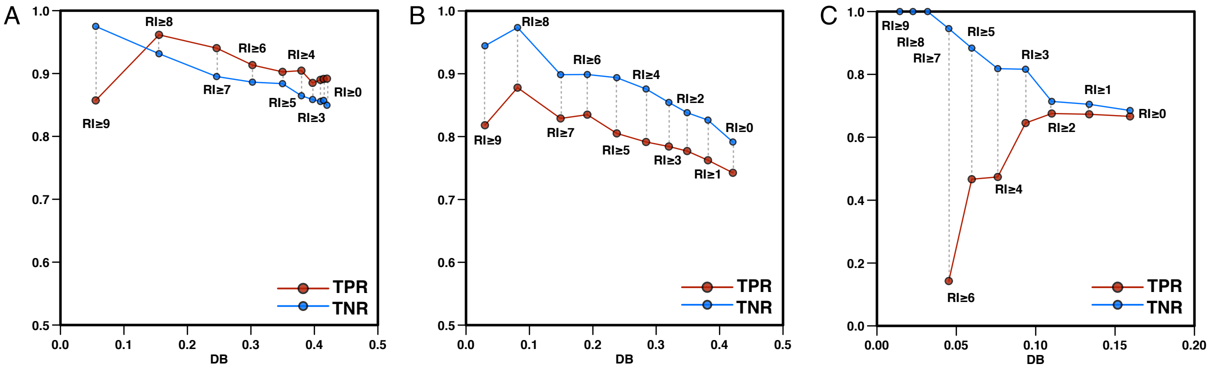

Fig. S1. Performance Meta-SNP as a function of the RI. Accuracy of Meta-SNP improves as a function of improving Reliability index (RI) on all NSV-2012 subsets (*Consensus* in panel A; *Majority* in panel B and *Tie* in panel C). Note that there are only 14, 11, and 31 disease causing variants at RI>=9, 9 and 3, resulting in an artifact of the curves - an unexpected drop in accuracy in panel A,B, and C respectively. TPR and TNR are defined in Methods. DB is the fraction of the NSV-2012 dataset with an RI higher or equal than a given threshold.
